# Characterization of 2D PLA Structural Metamaterials—Methodology and Challenges for Properties Assessment

**DOI:** 10.3390/polym18151832

**Published:** 2026-07-27

**Authors:** Ricardo Coelho, Teresa Abreu, Patrícia Freitas Rodrigues, Bernardo Alves, Daniel Gatões, Carlos Leitão

**Affiliations:** University of Coimbra, CEMMPRE, ARISE, Department of Mechanical Engineering, Rua Luis Reis Santos, 3030-788 Coimbra, Portugal

**Keywords:** mechanical characterisation, mechanical metamaterials, digital image correlation, wall thickness correction

## Abstract

Mechanical (or structural) metamaterials offer a paradigm shift in the mechanical response of advanced structures, achieving performance distinct from their base material through architected unit cells that geometrically tailor stress distribution. Characterising these structures demands methodologies capable of capturing localised deformations while reconciling numerical predictions with as-manufactured behaviour. This study introduces a Digital Image Correlation (DIC) based methodology for the mechanical characterisation of three two-dimensional PLA lattice metamaterials, produced by material extrusion (MEX) and selected from a computational screening of approximately 57,000 candidate geometries to span the negative, zero and positive Poisson’s ratio regimes. Predicted and measured Poisson’s ratios agreed to within 0.01 across all three geometries without correction, whereas structural rigidity deviated substantially from nominal geometry predictions. A targeted correction strategy, using micro-computed tomography (µCT) to measure wall thickness at high-stress concentration zones identified through finite element modelling, reduced these deviations from 46% to 7% for the auxetic geometry, and from 25% to 2% for the positive Poisson geometry. These results show that Poisson’s ratio and rigidity respond differently to manufacturing-induced dimensional deviation, and that efficient, targeted geometric correction, rather than full model reconstruction, is sufficient to reconcile numerical and experimental behaviour for these structures. This approach offers a practical framework for the experimental evaluation and numerical validation of mechanical metamaterials.

## 1. Introduction

In recent years, architected materials exhibiting unconventional mechanical responses, such as negative, zero, or even tailored Poisson’s ratios, have attracted growing attention due to their ability to decouple mechanical performance from intrinsic material properties and instead harness geometry to achieve bespoke behaviours [1,2,3,4,5,6,7]. Among the unusual mechanical behaviours, auxetic re-entrant structures are the most studied mechanical metamaterials. Characterised by a negative Poisson’s ratio, auxetic structures display lateral expansion when stretched and lateral contraction under compression, opposite to conventional materials, and can offer enhanced indentation resistance, increased shear stiffness, improved toughness, and superior energy absorption compared to conventional lattices under appropriate design and loading conditions. This behaviour arises from their specific micro- and meso-structural geometry. Materials with zero Poisson’s ratio, in contrast, maintain dimensional coherence under load, which is advantageous in precision engineering, vibration isolation, and sealing applications [8,9,10,11,12].

Additive manufacturing (AM), particularly material-extrusion-based (MEX) 3D printing, has emerged as a key enabler for the fabrication of such structures [13]. The geometric freedom offered by AM allows for the realisation of complex lattice architectures that would be difficult, or impossible, to manufacture using traditional methods, thereby facilitating systematic exploration of geometry-dependent mechanical responses across different Poisson regimes [14,15,16]. MEX is especially attractive due to its accessibility, material versatility, and suitability for polymer-based metamaterials. However, MEX relies on the deposition of discrete extrusion lines, which makes the fabrication of thin-wall and single-extrusion lattice struts highly sensitive to nozzle diameter, extrusion flow control, and slicing algorithms.

The geometric nature of the metamaterial responses demands a high level of dimensional accuracy. When pushing the limits of MEX towards thin-wall lattice production, the process becomes particularly sensitive to slicing algorithms and deposition paths, which often introduce deviations from nominal CAD geometry. Common defects include local wall-thickness variation, joint overbuild at node intersections, and discontinuities in extrusion [14,17,18,19,20]. These imperfections can lead to pronounced discrepancies between numerical predictions based on idealised geometries and experimentally measured responses [21,22].

Previous studies have investigated auxetic, zero-Poisson-ratio and conventional lattice systems using numerical modelling, compression testing and digital image correlation (DIC), demonstrating the ability of full-field measurement techniques to capture complex deformation mechanisms and Poisson kinematics under in situ loading. For example, McDonald et al. (2011) [20] used 3D X-ray microtomography combined with DIC to track local rib bending and node rotation in auxetic polyurethane foams during tensile loading, while Trippel et al. (2021) [21] employed global FE-based DIC to calibrate simplified beam-element models of SLM-manufactured antitetrachiral lattices, showing that DIC-derived displacement fields can significantly improve numerical fidelity. More recently, Atilla Yolcu and Okutan Baba (2022) [22] examined how the choice of measurement region and target points affects the experimentally determined Poisson’s ratio of 2D auxetic structures, highlighting the sensitivity of strain measurements to local kinematic heterogeneity. Akbay et al. (2024) [23] used DIC to characterise deformation in PLA lattice structures under torsion and compression, highlighting its effectiveness in capturing localised strain patterns in AM polymers. Similarly, Wang et al. (2023) [24] employed DIC to analyse the deformation of metal-ceramic hybrid auxetic lattices under quasi-static and dynamic loading, confirming its suitability for complex metamaterial architectures.

Despite these advances, a substantial proportion of numerical studies on AM lattices continue to rely on idealised CAD geometries, neglecting the geometric variability introduced during fabrication. This limitation is evident even in recent investigations: Eren et al. (2024) [25] evaluated the in-plane compression of Ti6Al4V auxetic lattices but noted that manufacturing-induced deviations, such as strut-thickness variation, significantly affected mechanical performance; however, these deviations were not fully integrated into the numerical models. Likewise, Li et al. (2024) [26] used X-ray computed tomography to quantify geometric defects in Ti6Al4V lattice structures and demonstrated that strut-thickness variation has a dominant influence on stiffness and strength, yet such corrections are rarely incorporated into FEM. These studies tend to treat geometric variability only partially or without coupling to full-field experimental data; this gap is particularly evident for thin-walled polymer lattices produced by MEX.

In this context, the present work combines in situ DIC with a targeted, specimen-specific correction of as-fabricated wall-thickness deviations at critical stress concentration zones, enabling a systematic quantification of their impact on both the global mechanical response and the predictive accuracy of finite element simulations, particularly with respect to stiffness and Poisson kinematics. This study systematically compares three representative 3D-printed polylactic acid (PLA) lattices engineered to exhibit negative, zero, and positive Poisson’s ratios. The methodology integrates MEX, full 3D finite element simulations, and compression testing supported by DIC to quantify deformation behaviour, stiffness, and Poisson kinematics.

The primary goal of this study is to develop and validate a DIC based experimental methodology for characterising the mechanical behaviour of 2D PLA lattice metamaterials produced by MEX, across the full Poisson’s ratio spectrum, with positive Poisson’s ratio lattices serving as a mechanical baseline for comparison against the auxetic and zero Poisson’s ratio regimes. In support of this goal, a large-scale computational screening was used to select three representative geometries spanning this spectrum, and a specimen specific correction of as-fabricated wall thickness deviations at critical stress concentration zones was developed to ensure that the numerical predictions used to validate the experimental methodology are themselves reliable. Together, these stages provide practical guidelines for enhancing simulation accuracy and manufacturing robustness in thin wall MEX metamaterials across the full spectrum of Poisson’s ratio regimes.

The novelty of this work presents itself as two main aspects. First, the candidate geometries themselves originate from an in-house generative and screening algorithm (Section 2.1) that guarantees fully connected, non-redundant and tessellatable unit cells, with structural feasibility further screened via Beam 1D simulation. This enables systematic selection of representative geometries across the full Poisson’s ratio spectrum from a design space of approximately 57,000 candidates. Second, the study introduces a closed-loop correction strategy in which preliminary 3D FEM identifies critical stress concentration zones, µCT measurement is targeted specifically to those zones rather than the full part, and the resulting as-built wall thickness data is fed back into a full 3D solid FEM validated directly against DIC. This contrasts with prior studies that have characterised as-fabricated geometric deviation using µCT [25,26] or calibrated simplified models against DIC [21], but have not closed the loop by feeding measured deviation back into a validated 3D solid FEM. The methodological contribution of this work lies not in the utilised tools individually, but in their systematic integration and in the specimen-specific correction strategy that couples the metrological data to the numerical models, providing practical guidelines for improving simulation fidelity of thin-walled MEX metamaterials.

## 2. Materials and Methods

**Figure 1:**
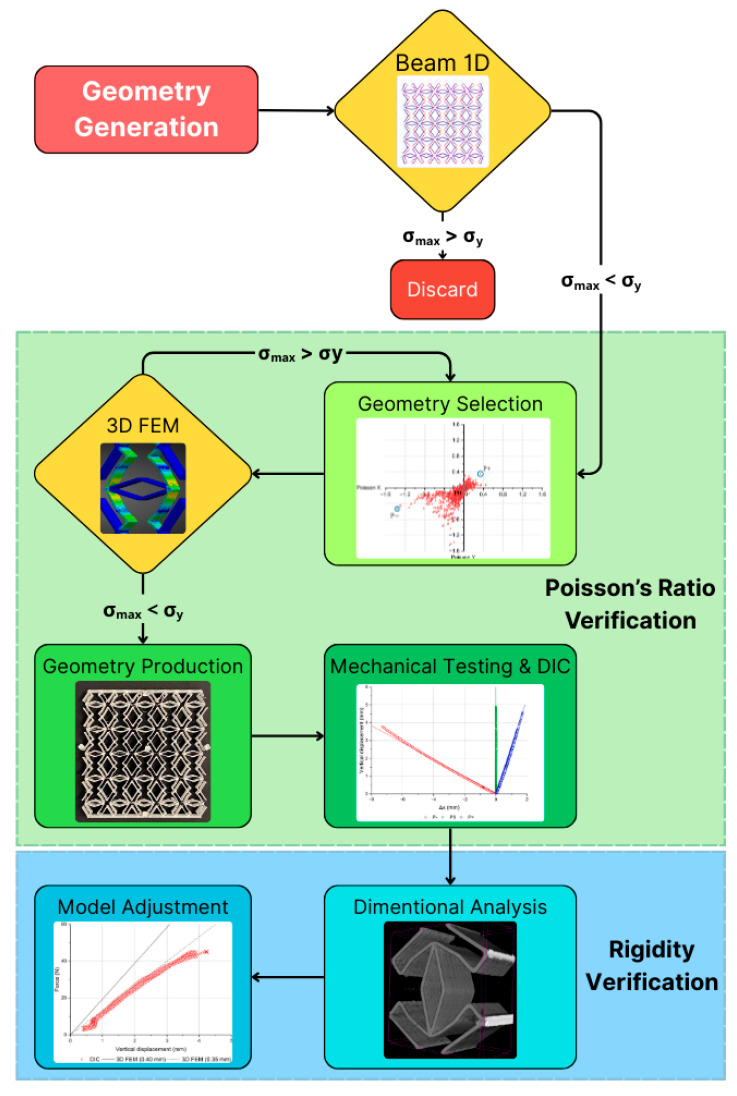
Workflow diagram.

This section describes the multi-stage methodology used to design, simulate, fabricate and characterise the mechanical metamaterials analysed in this study. The workflow, presented in Figure 1, combines a preliminary Beam 1D numerical screening to identify candidate geometries, followed by full 3D FEM to evaluate their structural response under compression. The selected geometries are then manufactured using MEX, and their dimensional accuracy is assessed using optical microscopy and micro-computed tomography to quantify wall-thickness deviations introduced during slicing and deposition. Finally, mechanical compression tests coupled with DIC are performed to measure the Poisson kinematics and rigidity of each geometry. The combination of these steps enables a direct comparison between numerical predictions and experimental behaviour and supports the subsequent 3D FEM re-parametrisation.

### 2.1. Simulation and Selection

Possible geometries were designed using a proprietary in-house Python (version 3.14.2) code developed at the University of Coimbra, which generates strut networks by connecting the nodes of a regular point grid. A candidate configuration is accepted as a valid unit cell only if it satisfies a strict set of geometric-validity rules:

(i) The network must be fully connected, with no isolated members;
(ii) Dangling struts are not permitted;
(iii) The network must contact all four boundaries of the unit-cell domain, ensuring load transfer between adjacent cells and valid periodic tessellation;
(iv) Fourfold mirror symmetry is imposed about the two central axes, after which member intersections are recomputed, and connectivity and feasibility are re-validated on the complete cell;
(v) Duplicate configurations are identified and discarded, ensuring that each generated unit cell is unique.

After generating circa one million different geometries, each unit cell was systematically evaluated as one-dimensional beams (Beam 1D) in ANSYS MAPDL, in order to minimise computational demand. The simulations calculated the structural response to an imposed displacement of 5% in both the X and Y directions, enabling the determination of Poisson’s ratio, von Mises stress, and reaction forces along each axis. For the model, a square beam was utilised, with a size of 0.4 mm, E = 2.94 GPa and ν = 0.33. Poisson values were calculated by dividing the averaged nodal displacements along the left side of the structure, by half of the imposed vertical displacement. Only geometries exhibiting von Mises stress values below the yield strength of PLA (50 MPa) were retained for further analysis. Reaction forces and von Mises stress were used as feasibility filters at this stage, ensuring that all candidate geometries would remain within the elastic regime under the imposed displacement. Poisson’s ratio was subsequently adopted as the sole selection criterion for the final three geometries, since it is the property that defines the mechanical categories under investigation (negative, zero and positive) and directly determines the deformation mechanism to be characterised. Rigidity was not treated as a selection input but was instead used as a validation target in the subsequent 3D FEM and experimental stages.

Following completion of all simulations, the results were analysed to identify geometries with the desired mechanical characteristics. For this study, three configurations were selected because they represent the extreme cases within the design space: one with a negative Poisson’s ratio (P−), one with a Poisson’s ratio of zero (P0), and one with a positive Poisson’s ratio (P+). These three geometries were chosen as representative configurations to enable a systematic comparison of the mechanical behaviour across the full Poisson spectrum.

The selected geometries were then modelled, and 3D FEM numerical simulations were performed using the ANSYS software version 2024 R3 (ANSYS Inc., Canonsburg, PA, USA). Each unit cell was designed with dimensions of 16 × 16 × 10 mm and a wall thickness of 0.4 mm. The unit cells were subsequently arranged in a 5 × 5 matrix, resulting in an overall structure size of 80 × 80 × 10 mm.

While a 5% deformation (4 mm) was applied in the Beam 1D simulations, the 3D FEM analysis employed a total deformation of 10% (8 mm), applied in 1 mm incremental steps on the top surface of the structure, while the bottom surface was fully constrained. This approach was adopted to investigate the structure’s behaviour beyond the elastic domain. For the Poisson’s ratio calculation, the length deviation between two nodes, chosen to match the DIC reference line, was divided by the imposed vertical displacement.

The geometry was discretised using quadratic finite elements, with a target element edge size of 1 mm, resulting in 97,331 elements. To verify the independence of the results from the discretisation, a mesh convergence study was performed on the three selected geometries, with element edge sizes ranging from 2.0 mm to 0.5 mm. The reaction force varied by less than 7% between the adopted 1 mm mesh and the finest mesh tested. Anisotropic material behaviour was not considered in this study. Anisotropy in MEX-printed parts is predominantly governed by interlayer bonding weakness along the build direction, rather than by in-plane variation, and since the build direction here corresponds to the specimen thickness, the structures were predominantly loaded along the axis least affected by this mechanism.

### 2.2. Structure Production

The selected structures were manufactured using a Prusa Mk4 3D printer equipped with a 0.40 mm nozzle and a layer height of 0.20 mm. Slicing was performed with the Orca Slicer software (version 2.3.2).

Polylactic acid (PLA) was adopted due to its accessibility, biodegradability, and stable printing behaviour, making it a preferred material for this type of research and prototyping [27,28,29].

**Figure 2:**
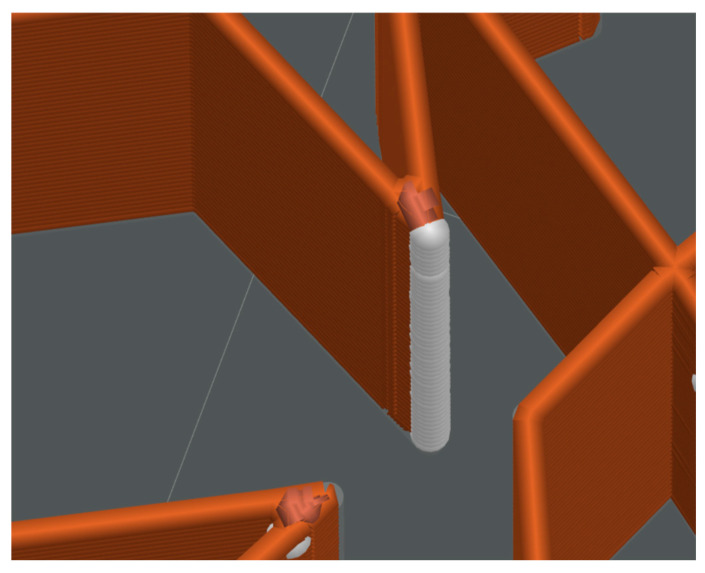
Sliced segment of a structure with structure lines (in orange), seam placement (in white) and ironing (in dark orange).

Several adjustments were made to the printing parameters to ensure the structural integrity, as the truss walls consisted of only a single extrusion line. One such adjustment was the elimination of the seam gap in combination with aligned seams, ensuring that the nozzle completed each loop at its starting point, eliminating the possibility of gaps occurring between the end and start of the line, thus producing stronger joints between trusses. Additionally, the ironing technique [30] was applied between layers to fill potential voids in the joints with material. Figure 2 illustrates these techniques, with the white points being the seam locations and the red zones the areas where ironing was applied.

Another peculiarity of these types of single extrusion wall structures is that since the wall thickness of the CAD model and the nozzle diameter are the same, the classic wall generation algorithm of the slicers has difficulties creating the gcode during the slicing. To overcome this limitation, the Arachne algorithm was used, which uses adaptable extrusion widths, widening or thinning the extrusion width by back pressure compensation [31]. However, due to the way this algorithm adapts extrusion paths, the resulting line width may deviate from the nominal value specified in the slicing parameters. In these structures, such variations translate directly into deviations of the actual wall thickness from the intended design. Consequently, these thickness variations introduce uncertainty in the predicted rigidity from the 3D FEM simulations.

### 2.3. Dimensional Characterisation

Dimensional characterisation was carried out to quantify the deviations between the nominal wall thickness (0.4 mm) and the actual thickness of the printed structures, since such deviations strongly influence the stiffness of lattices.

An initial qualitative assessment of geometric uniformity was performed using optical microscopy (OM) with a LEICA DM4 binocular microscope equipped with an integrated video camera. Images were acquired at 50× magnification. This preliminary inspection revealed noticeable variations in wall thickness across the struts. To obtain accurate thickness measurements, a micro-computed tomography (µCT) scan was subsequently carried out on a unit cell of each structure using a Bruker SKYSCAN 1275 system operating at 50 kV and 200 µA. Image acquisition was performed in step-and-shoot mode, without a filter, a rotation step of 0.4°, and a minimal exposure time of 35 ms. The resulting pixel size was 25 µm. After image reconstruction, an automatic OTSU thresholding procedure was applied, and both localised thickness values and thickness distributions were computed for each structure. These measurements provided the basis for evaluating how manufacturing-induced variability influences the resulting mechanical response.

### 2.4. Mechanical Testing

**Figure 3:**
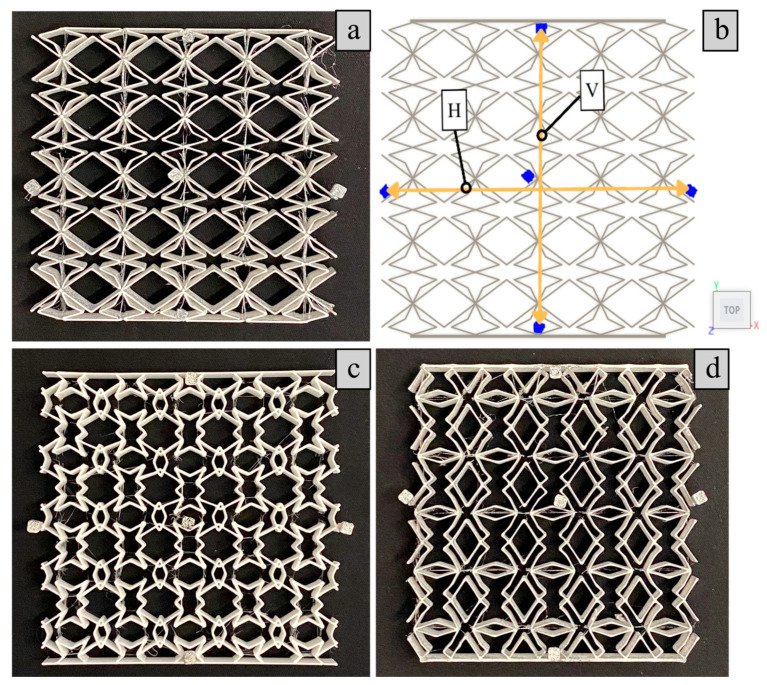
DIC reference markers and measurement lines: (**a**) P0 specimen with attached reference pins; (**b**) CAD model showing horizontal and vertical measurement lines; (**c**) P+ specimen with attached reference pins; (**d**) P− specimen with attached reference pins.

In order to quantify the Poisson kinematics and stiffness of the geometries, a series of compression tests were performed using a Shimadzu AGS-100kNX universal testing machine. To evaluate the consistency of the structure’s elastic properties, five load cycles with a 4 mm stroke were performed, followed by a final 8 mm stroke cycle to assess the elastic limit of the structures. All cycles were conducted at a downstroke speed of 2 mm/min and an upstroke speed of 4 mm/min. Simultaneously, a 3D Digital Image Correlation (DIC) analysis was performed using the ARAMIS software (Carl Zeiss GOM Metrology GmbH, Braunschweig, Germany). However, due to the small thickness of the lattice walls of approximately 0.4 mm, the software was unable to reliably detect the surface of the structure. To overcome this limitation, several pins with an area of 3 × 3 mm^2^ were attached in zones that did not impact the structure’s deformation to act as reference nodes (Figure 3a). These nodes enabled the definition of reference lines (Figure 3b) which the software could accurately measure the length deviations for each compression stage, thus obtaining the vertical and horizontal displacements. The three-dimensional DIC configuration was employed to also monitor out-of-plane displacement throughout the compression cycles. Out of plane displacement was recorded, with the largest value being 7% of the imposed 4 mm vertical stroke, observed for P−.

The typical parameters used to calculate the Poisson’s ratio are the lateral and longitudinal strains ($\upsilon =-\varepsilon_{x}/\varepsilon_{y}$); however, since $\varepsilon =\Delta l/l_{0}$, where Δl is the length variation in a direction and l0 is the initial size in the same direction, due to the square nature of the structure in XY, lx0 = ly0. Additionally, since the compression is unidirectional, ${\Delta l}_{y}=-\Delta y$, where Δy is the imposed vertical displacement. These deductions are shown in Equation (1).

(1) $$\upsilon =-\frac{\varepsilon_{x}}{\varepsilon_{y}}=-\frac{\frac{{\Delta l}_{x}}{l_{x0}}}{\frac{{\Delta l}_{y}}{l_{y0}}}=\frac{{\Delta l}_{x}}{\Delta y}$$

## 3. Results

### 3.1. Geometry Selection

**Figure 4:**
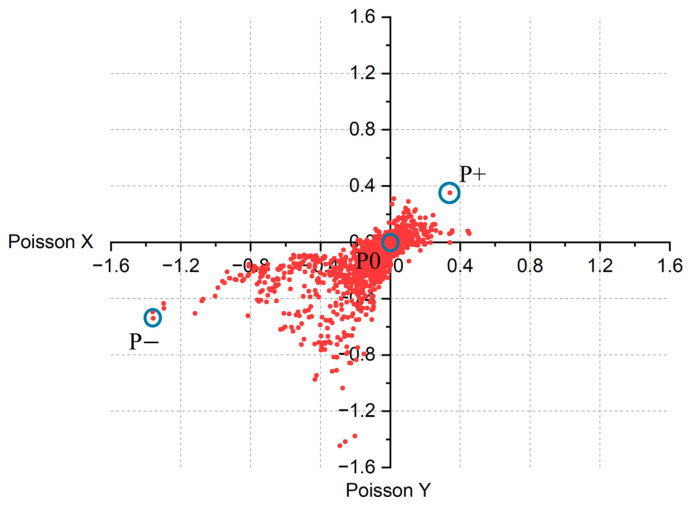
Poisson ratio distribution.

Figure 4 presents the design space of the candidate geometries, where each point corresponds to a distinct configuration. This design space of pre-selected structures consisted of 56,985 geometries, with Poisson values ranging from −1.45 to 0.35, with each point indicating a distinct configuration.

As previously mentioned, three geometries were selected for this study (Figure 4). These were chosen due to their representation of the most extreme cases within the design space, while taking into consideration the printability of the structures. This selection strategy was intended to test the proposed characterisation methodology across the full range of achievable mechanical behaviours, rather than to target a specific application, since the extremes of the design space correspond to the most pronounced deformation mechanisms and therefore represent the most demanding cases for validating the DIC-based approach. The unitary cell and initial Poisson estimate from these simulations are presented in Table 1.

**Figure 5:**
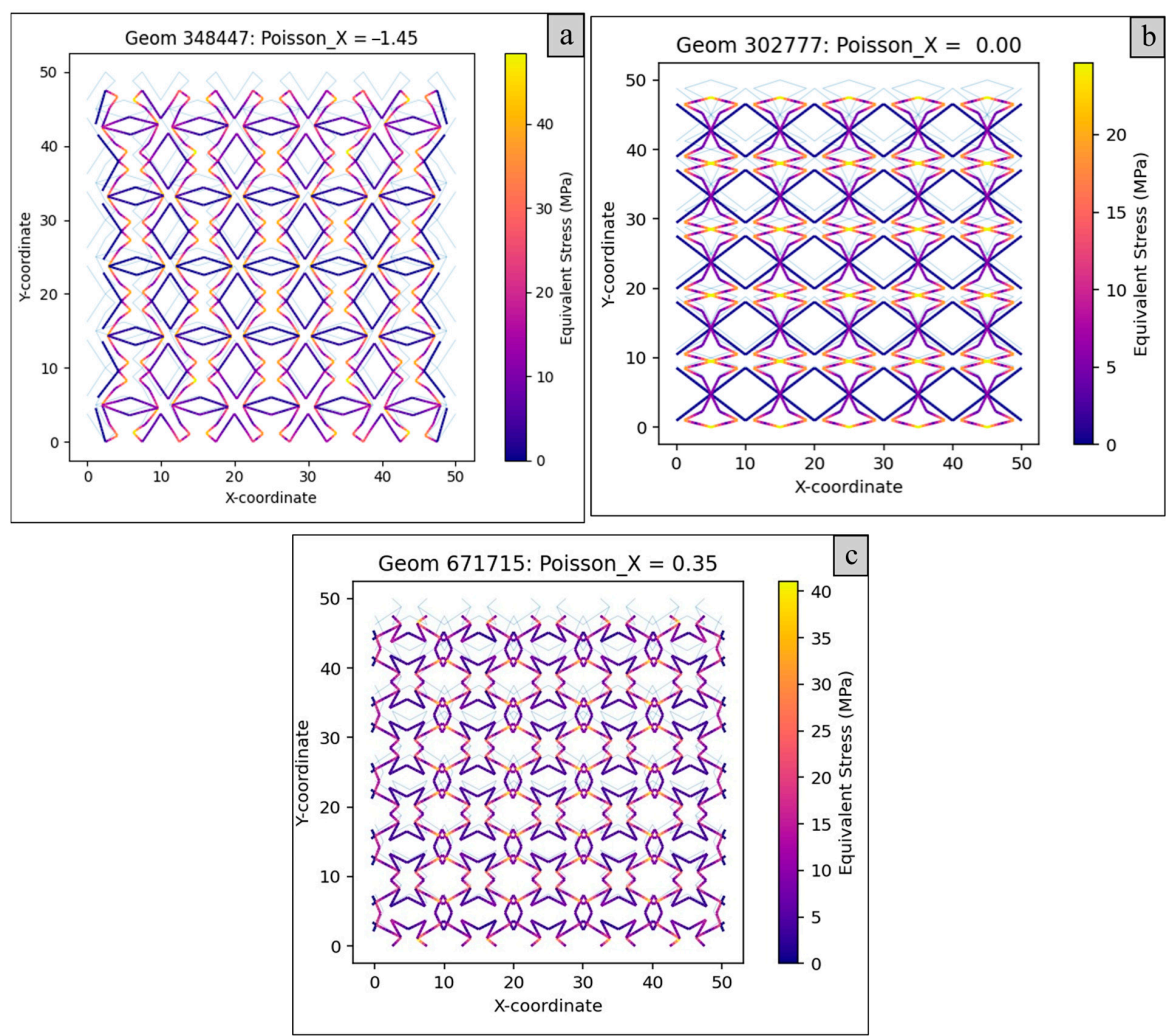
Beam 1D von Mises stress distribution for (**a**) P− geometry, (**b**) P0 geometry and (**c**) P+ geometry.

Beam 1D stress distributions for the selected geometries were evaluated (Figure 5a–c). All configurations exhibit their highest equivalent stresses at node junctions, corresponding to the closest points which are connected to the elements with the highest bending, as expected. This stress concentration supports the reinforcement strategies adopted during MEX additive manufacturing (Section 2.2), particularly the seam-control and ironing adjustments implemented to minimise local thinning.

**Figure 6:**
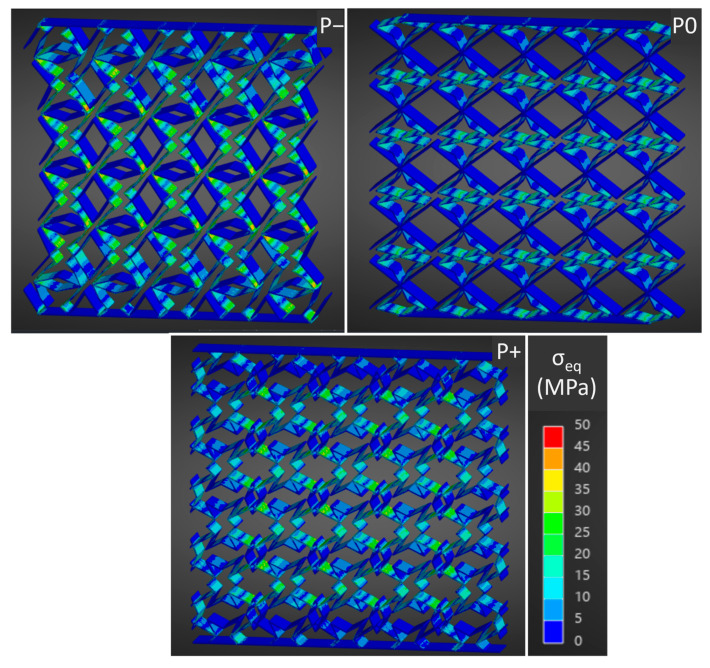
Predicted von Mises stress field (at 5% deformation).

**Figure 7:**
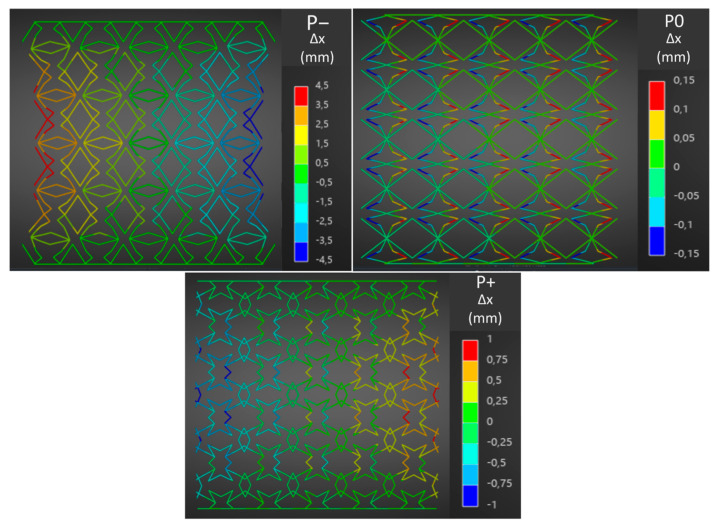
Predicted horizontal displacements (at 5% deformation). Colour scales are independent for each geometry due to the substantially different magnitude of horizontal displacement associated with each Poisson’s ratio regime.

For a more precise evaluation of the selected structures, the 3D FEM simulation was performed. Figure 6 provides the von Mises stress distributions and Figure 7 provides the horizontal displacements (Δx) of all the geometries. The stress results show a close alignment with the previous Beam 1D simulations, also indicating that the zones with the greatest stress concentration are at the lattice connection points. Additionally, P− showed stress values close to the yield stress, indicating some likelihood of plastic deformation at the end of the 4 mm stroke compression cycles.

Having all the numerical simulation data available, the structures were then 3D printed and experimentally tested to validate the numerical predictions.

### 3.2. Experimental

**Figure 8:**
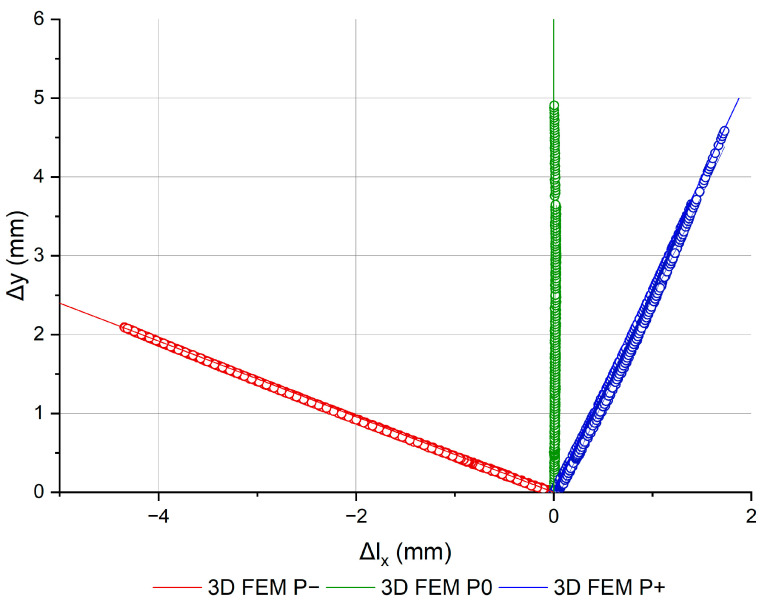
Vertical displacement and horizontal length deviation (Δx) results from DIC (dots) and 3D FEM (lines).

The Δy and corresponding Δl_x_, obtained from DIC measurements and 3D FEM simulations for each structure can be observed in Figure 8. The 3D FEM results presented in Figure 8 correspond to the original model, using the nominal wall thickness of 0.4 mm, with no adjustment performed against the experimental data. Only the stages in the elastic regime were considered.

The Poisson’s ratio values obtained by 3D FEM and DIC were derived from a linear fit across all loading cycles performed on each specimen and are presented in Table 2. Predicted (3D FEM) and measured (DIC) Poisson’s ratio agreed closely across all three geometries, within 0.01 in absolute terms for P− and P0 and matching to two decimal places for P+. For the experimental Poisson, the standard error of the fitted slope through the inverse relation in Equation (1) indicates that the calculated Poisson’s ratio was tightly constrained across repeated cycles for all three geometries. The comparatively larger relative uncertainty in the fitted slope for P0 reflects its near-zero horizontal displacement by design, rather than reduced experimental consistency, since a small absolute scatter represents a much larger fraction of a near-null signal than of the substantially larger displacement ranges observed for P− and P+. The coefficient of determination (R^2^) for these fits was 0.996 for P+ and 0.999 for P−, indicating high internal consistency of the underlying displacement measurements. For P0, R^2^ was 0.522, with the same near-null slope effect described above being responsible for the lower value. These R^2^ values characterise the internal consistency of the DIC linear fits and should not be interpreted as a statistical comparison against the FEM predictions.

The Beam 1D method produced more conservative Poisson values, primarily because the procedure used to calculate Δlx differs from that employed in the other methods (see Section 3.4).

**Figure 9:**
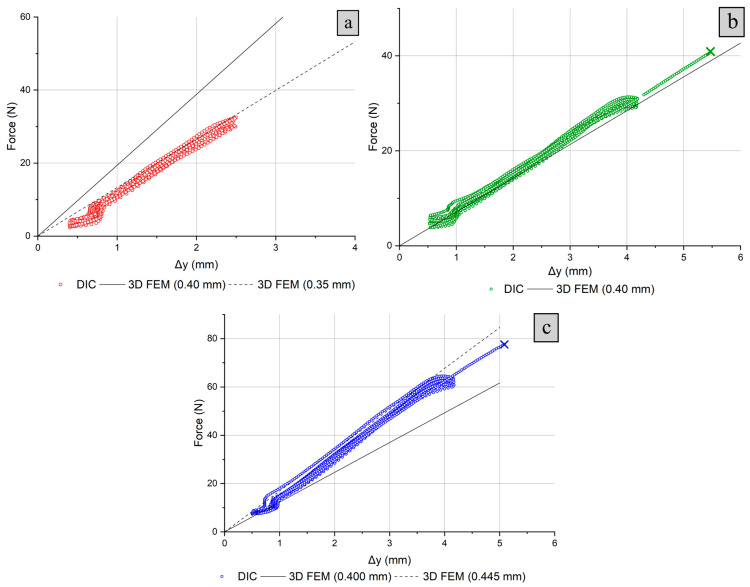
Force-vertical displacement results from DIC (dots) and 3D FEM simulations with original model (line) and adjusted model (dashed line) of (**a**) P−, (**b**) P0 and (**c**) P+.

Figure 9a–c presents the relation between the imposed vertical displacement and the reaction force for structures P−, P0 and P+, respectively. DIC data (dots) and 3D FEM simulations (lines) deviate from the expected response. Since this deviation can be largely attributed to truss behaviour, a wall thickness adjustment was performed to match the manufactured structure (dashed lines). Wall thickness measurements on printed structures are shown in Section 3.3.

The results indicate that in P0 and P+, plastic deformation (indicated by the x on the graphs) occurred only on the final 8 mm displacement cycle; however’ P− (Figure 9a) entered the plastic deformation regime around 2.5 mm of vertical displacement. Since the focus of this study is the application of these structures within the elastic regime, only the elastic stages were considered for the rigidity calculations, consistent with the approach used for the Poisson’s ratio evaluation.

### 3.3. Dimensional Analysis

**Figure 10:**
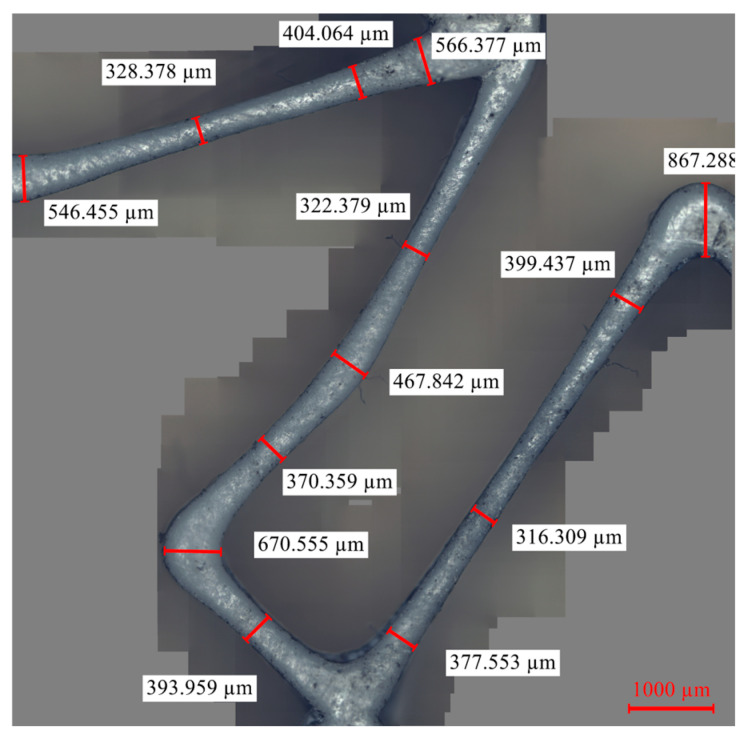
OM image reconstruction of 1/4 of the P− unit cell.

The geometrical accuracy of the structures was evaluated using OM (Figure 10). Preliminary measurements show evidence that the wall thickness was not constant along the truss. Measurements were performed on all geometries, and the same inconsistencies were observed. The inconsistencies can be a direct consequence of the use of Arachne algorithm, which largely influences extrusion flow rate, as described in Section 2.2.

**Figure 11:**
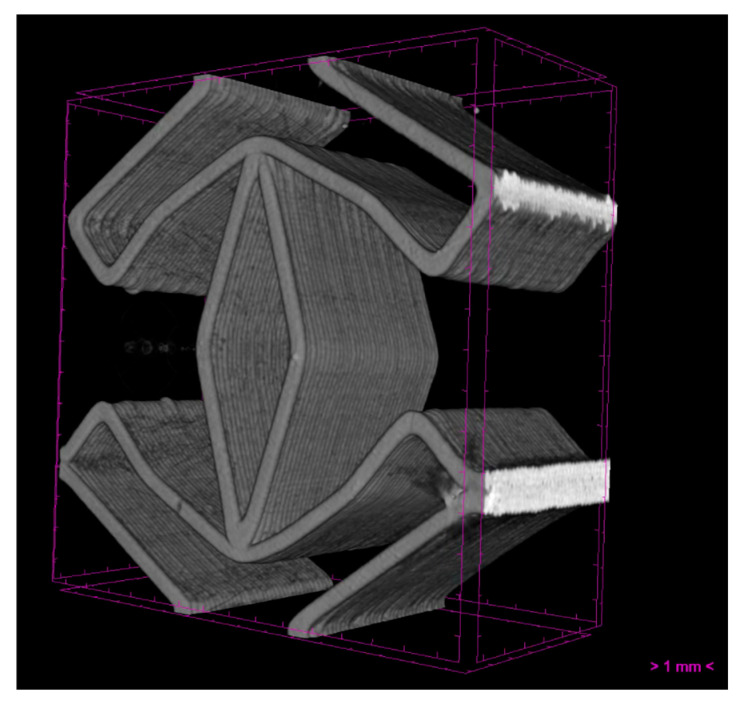
µCT visualisation of the P− unit cell.

Optical microscopy is unreliable for determining the thickness throughout the entire volume of the truss walls, as it can only measure the topmost extrusion line, while underlying layers may be thinner. To accurately quantify thickness deviations, µCT images of a representative unit cell were acquired for each geometry (Figure 11 shows P− as an example).

**Figure 12:**
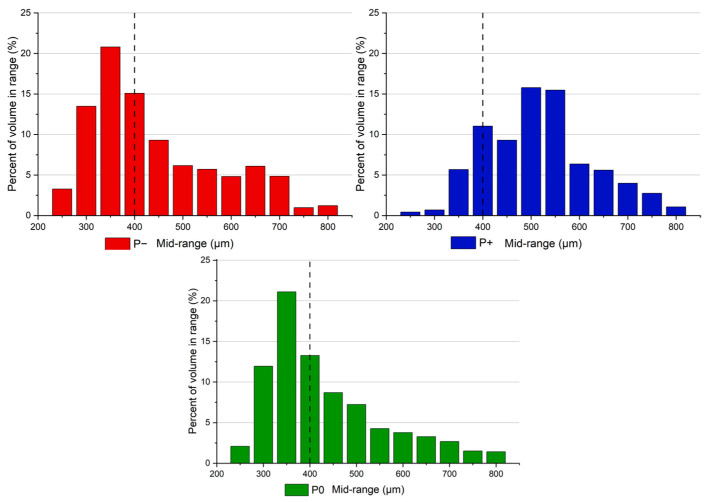
Wall thickness distributions (dashed lines indicate nominal wall thickness).

After the thresholding, the wall thickness of the structure was calculated. The resulting thickness distribution of the structures is represented in Figure 12.

As observed, wall thickness varies significantly throughout the structure. Therefore, averaging these values does not yield a reliable model for stiffness predictions. This may be attributed to the disproportionate mechanical influence of the slender lattice struts relative to the thicker joints. The auxetic mechanism of re-entrant structures in the elastic regime is primarily governed by strut flexure. Consequently, the influence of thicker joints on overall stiffness is relatively low.

**Figure 13:**
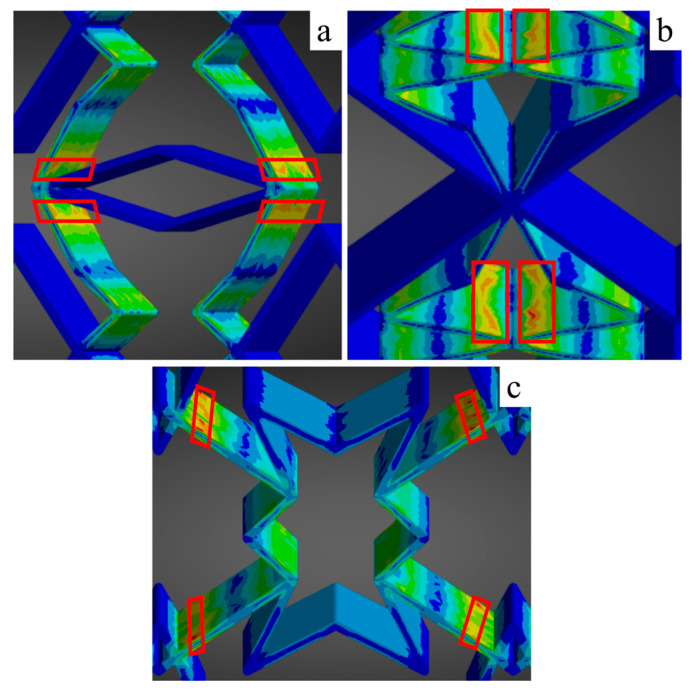
Von Mises stress distribution in the unit cell of (**a**) P−, (**b**) P0 and (**c**) P+. Measurement volumes highlighted in red.

Since averaging the thickness measured in µCT proved to be unreliable in obtaining a more representative model, another strategy was pursued. The highest stress concentration zones in the 3D FEM simulations (red rectangles in Figure 13) were measured in µCT, and another model was created with a constant wall thickness of the average measured values for each geometry.

**Figure 14:**
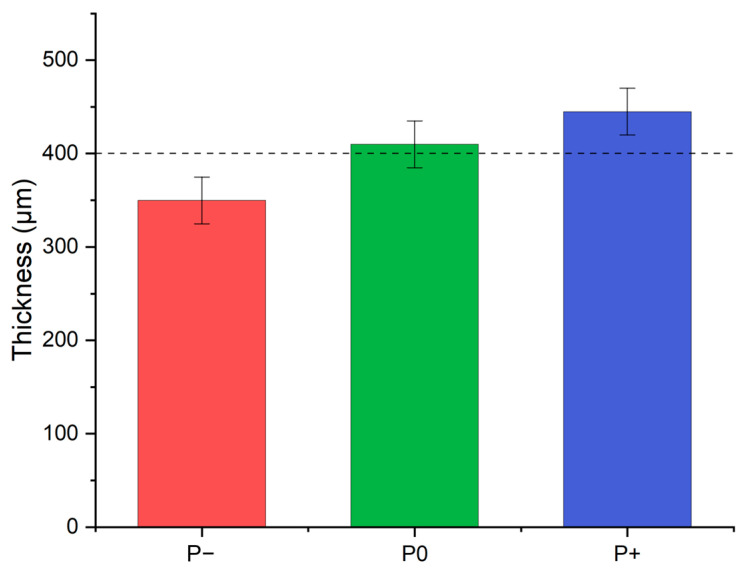
Wall thickness measurements from µCT (dashed line indicate nominal wall thickness).

The measured values are represented in Figure 14. These results are consistent with the deviations observed in Figure 9: P− exhibited a reduced thickness, leading to lower-than-expected rigidity, whereas P+ showed an increased thickness and consequently higher-than-expected rigidity. P0 displayed the closest agreement with the predicted rigidity, with only a 6.6% deviation between the simulated and experimental values (Table 3), and since the measurement error crossed the nominal 400 µm value, the CAD model adjustment was not performed for this geometry.

The numerical models for P− and P+ were subsequently adjusted to match their measured thicknesses, namely 350 µm and 445 µm, respectively. The resulting force–displacement responses from the updated simulations are shown as black dashed lines in Figure 9a,c. The rigidities obtained from the linear fits of the revised 3D FEM simulations, together with those derived from the DIC measurements, are summarised in Table 3.

A spatially resolved correction, reconstructing the full as-fabricated geometry directly from the µCT dataset, would in principle be achievable given the voxel resolution of 25 µm relative to the measured wall thicknesses, but would require a substantially more complex segmentation and mesh-reconstruction workflow. Since rigidity in these structures is governed predominantly by strut flexure at high-stress concentration zones rather than by the bulk average thickness, a constant thickness correction targeted at those zones reduced the deviation between predicted and experimental rigidity from 45.9% to 7.2% for P− and from 25.2% to 2.6% for P+, indicating that this simplified approach is sufficiently accurate for the purposes of this study without the added complexity of full geometric reconstruction.

### 3.4. Discussion

Beam 1D analysis provides an efficient first stage screening tool, identifying feasible geometries, extreme Poisson behaviours, and critical stress zones to be considered for subsequent 3D FEM and production stages. Although the Poisson’s ratio obtained from these simulations is useful for differentiating candidate geometries, it proved less accurate than the 3D FEM and DIC results. This discrepancy arises from the different displacement sampling methods used in each approach. Beam 1D Poisson values were obtained by averaging nodal horizontal displacement across the entire left edge of the structure, while 3D FEM values were obtained from a single node pair at mid-height, matching the DIC reference line H. Under the parabolic lateral displacement profile produced by the grip boundary conditions, averaging edge displacements reduces the larger mid-height displacement, due to the constrained top and bottom rows, reducing the calculated Poisson’s ratio. This was identified after the Beam 1D screening had already been completed across the full design space, so the screening was not re-run, since its role was to enable relative differentiation between candidate geometries ahead of 3D FEM and experimental validation rather than to provide an absolute quantitative prediction.

The present data allow a distinction to be drawn between the sensitivity of Poisson’s ratio and structural rigidity to localised printing defects. Poisson’s ratio predicted by the original 3D FEM model agreed with DIC measurements to within 0.01 across all three geometries, despite wall thickness varying meaningfully across each structure. By contrast, structural rigidity deviated from the same uncorrected model by 45.9% for P− and 25.2% for P+, and required correction based on measured wall thickness at critical stress zones to bring this deviation down to 7.2% and 2.6% respectively. This indicates that the kinematic response captured by Poisson’s ratio is comparatively insensitive to the localised dimensional variability observed in these structures, while rigidity is not.

DIC analysis proved to be a reliable tool for measuring the actual mechanical behaviour of the structures. While experimental results validated numerical predictions for Poisson’s ratio, rigidity values deviated significantly, revealing inconsistencies in lattice thickness. This highlights a critical consideration when applying this methodology to design and implement these types of metamaterials, which is the impact of the dimensional inaccuracies that emerge from the manufacturing process, which the simulations are currently incapable of predicting a priori.

By using a high-precision measurement technique like the µCT, it was possible to quantify these dimensional deviations. Also, by measuring only the regions of the unit cell with the highest stress identified through simulations, it was possible to adjust the models to yield more realistic values. This demonstrates that, for the geometries and manufacturing process investigated here, targeted correction of high-stress concentration zones is sufficient to substantially improve simulation accuracy, without requiring complex CAD modifications to reconstruct the full as-fabricated geometry. Whether this holds for other cell topologies or 3D lattice architectures has not been established in this study and would require independent validation.

In particular, extension to three-dimensional spatial architectures would first require establishing whether such structures exhibit a similarly tractable number of identifiable high-stress zones, since the present correction strategy relies on rigidity being governed by a small number of such zones rather than a distributed stress field.

Regarding extension to other materials and processes, preliminary evidence supports the generality of the underlying approach. In a related study by the authors on a two-dimensional auxetic Ti6Al4V metamaterial produced by laser powder bed fusion, calibrating the numerical model to as-built strut thickness reduced the deviation between predicted and experimental stiffness from 42% to 5% [32], a magnitude of correction consistent with the 45.9% to 7.2% and 25.2% to 2.6% reductions reported here for MEX-printed PLA. This suggests that model correction based on wall thickness may not be specific to the present material and process, though this remains a hypothesis based on analogy between two distinct material and process combinations rather than a confirmed generalisation, and would require dedicated experimental verification on additional materials and processes to establish.

These findings position the present methodology relative to existing DIC, µCT and FEM approaches applied to lattice metamaterials. McDonald et al. [23] combined µCT and DIC to track rib bending and node rotation in polyurethane foam, but did not feed measured geometric deviation back into a numerical model. Trippel et al. [24] used DIC to calibrate simplified beam-element representations of SLM metal lattices, without reference to µCT measured wall thickness or full 3D solid geometry. Li et al. [29] and Eren et al. [28] used CT to identify strut-thickness variation as a dominant influence on the stiffness and strength of Ti6Al4V lattices, but noted that such corrections were not incorporated into their numerical models. The present results extend this body of work by closing that loop for MEX-printed polymer lattices, demonstrating that a targeted, computationally efficient correction, rather than a full geometric reconstruction, is sufficient to bring predicted rigidity into close agreement with experimental measurements, while also showing that Poisson’s ratio, unlike rigidity, is comparatively insensitive to the same dimensional variability. A direct quantitative comparison of correction accuracy against these studies is not meaningful given the differing materials, element formulations and reported metrics involved, but the present approach complements them by demonstrating a complete, validated correction pathway for a material and process not covered by these prior works.

A limitation of the present study is that only one specimen was manufactured and tested per geometry. Also, since the compression tests were conducted to the elastic limit and beyond, resulting in irrecoverable plastic deformation, repeated testing of the same specimen was not possible. Repeatability of the elastic response within each specimen was assessed through five elastic deformation cycles prior to the final plastic deformation cycle, which showed consistent behaviour across cycles. However, inter-specimen variability arising from manufacturing was not captured in this study. Future work should include multiple specimens per geometry to quantify this source of variability.

## 4. Conclusions

This study successfully established and validated a robust methodology for the mechanical characterisation of two-dimensional mechanical metamaterials produced via MEX. By investigating three distinct lattice geometries across the negative, zero, and positive Poisson’s ratio regimes, the research demonstrated how structural architecture alone can be used to tune global stiffness and deformation mechanisms.

The key findings and contributions of this work are summarised as follows:

- Poisson’s ratio predictions from the original, uncorrected 3D FEM model closely matched DIC measurements across all three geometries (P− with −2.09 predicted vs. −2.08 measured, P0 with 0.00 vs. 0.01 and P+ with 0.38 vs. 0.38), confirming that geometry alone, without wall-thickness correction, is sufficient to predict Poisson’s ratio accurately for these structures.
- Structural rigidity, by contrast, deviated substantially from the original model, by 45.9% for P− and 25.2% for P+, due to wall-thickness variation introduced by the Arachne slicing algorithm. Targeting µCT measurement to the high-stress concentration zones identified through preliminary 3D FEM, rather than reconstructing the full as-fabricated geometry, was sufficient to correct this, with the resulting adjusted models reducing the rigidity deviation to 7.2% for P− and 2.6% for P+. Extending this approach to more geometrically complex spatial metamaterials, other cell topologies, or materials and processes with different dominant sources of dimensional deviation remains to be demonstrated.
- Greater manufacturing control and optimised printing parameters remain necessary for consistent wall thickness in engineering applications of these structures. Furthermore, the implementation of machine-learning tools to predict and compensate for dimensional inaccuracies during the design phase represents a promising avenue for future research.

It is important to note that the correction strategy proposed here does not require exhaustive µCT inspection of every manufactured part. Instead, high-stress concentration zones are first identified through preliminary 3D FEM, and µCT measurement is subsequently targeted only at these regions, substantially reducing the inspection burden relative to full-part scanning. The efficiency of this approach at production scale depends on the manufacturing process being sufficiently consistent that the identified deviation, in this case wall-thickness variation from the Arachne slicing algorithm, behaves predictably across parts rather than requiring re-characterisation for each individual structure. This work establishes the diagnostic and correction methodology and demonstrates it on three representative cases. Confirming the consistency of the underlying process deviation across a broader production run, and thereby the methodology’s applicability at scale, is left for further work.

Overall, this combined experimental-numerical approach offers a valuable framework for the informed design and optimisation of advanced architected materials, bridging the gap between idealised numerical predictions and the realities of additive manufacturing.

## Figures and Tables

**Table 1:** Selected geometries and Poisson values (from Beam 1D).

**Geometry**	**Unit Cell**	**Poisson (Beam 1D)**
P−	** 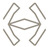 **	−1.45
P0	** 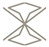 **	0.00
P+	** 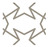 **	0.35

**Table 2:** Poisson’s ratio of the structures (acquired from linear fit of DIC and 3D FEM results).

Geometry	Beam 1D	3D FEM	DIC *
P−	−1.45	−2.09	−2.08 ± 0.0015
P0	0.00	0.00	0.01 ± 0.0002
P+	0.35	0.38	0.38 ± 0.0008

* Uncertainty reported for DIC values reflects the standard error of the linear fit across all loading cycles for the single tested specimen per geometry and represents fit precision rather than total measurement uncertainty.

**Table 3:** Rigidity results from 3D FEM and experimental testing.

	Thickness (µm)	Rigidity (N/mm)	Deviation (%)
P−	400	19.42	45.9
	Experimental	14.35	-
	350	13.31	7.2
P0	400	7.11	6.6
	Experimental	7.61	-
P+	400	12.34	25.2
	Experimental	16.49	-
	445	16.92	2.6

## Data Availability

Data is contained within the article.
